# Genome-Wide Association Mapping for Agronomic and Seed Quality Traits of Field Pea (*Pisum sativum* L.)

**DOI:** 10.3389/fpls.2019.01538

**Published:** 2019-11-26

**Authors:** Krishna Kishore Gali, Alison Sackville, Endale G. Tafesse, V.B. Reddy Lachagari, Kevin McPhee, Mick Hybl, Alexander Mikić, Petr Smýkal, Rebecca McGee, Judith Burstin, Claire Domoney, T.H. Noel Ellis, Bunyamin Tar'an, Thomas D. Warkentin

**Affiliations:** ^1^Crop Development Centre, Department of Plant Sciences, University of Saskatchewan, Saskatoon, SK, Canada; ^2^AgriGenome Labs Pvt. Ltd, Hyderabad, India; ^3^Department of Plant Sciences and Plant Pathology, Montana State University, Bozeman, MT, United States; ^4^Crop Research Institute/Department of Genetic Resources for Vegetables, Medicinal and Special Plants, Olomouc, Czechia; ^5^Forage Crops Department, Institute of Field and Vegetable Crops, Novi Sad, Serbia; ^6^Department of Botany, Palacký University, Olomouc, Czechia; ^7^Grain Legume Genetics and Physiology Research Unit, USDA, ARS, Pullman, WA, United States; ^8^INRA, UMRLEG, Dijon, France; ^9^Department of Metabolic Biology, John Innes Centre, Norwich, United Kingdom; ^10^School of Biological Sciences, University of Auckland, Auckland, New Zealand

**Keywords:** field pea, genetic diversity, genome-wide association study, genotyping-by-sequencing, single nucleotide polymorphisms

## Abstract

Genome-wide association study (GWAS) was conducted to identify loci associated with agronomic (days to flowering, days to maturity, plant height, seed yield and seed weight), seed morphology (shape and dimpling), and seed quality (protein, starch, and fiber concentrations) traits of field pea (*Pisum sativum* L.). A collection of 135 pea accessions from 23 different breeding programs in Africa (Ethiopia), Asia (India), Australia, Europe (Belarus, Czech Republic, Denmark, France, Lithuania, Netherlands, Russia, Sweden, Ukraine and United Kingdom), and North America (Canada and USA), was used for the GWAS. The accessions were genotyped using genotyping-by-sequencing (GBS). After filtering for a minimum read depth of five, and minor allele frequency of 0.05, 16,877 high quality SNPs were selected to determine marker-trait associations (MTA). The LD decay (LD_1/2max,90_) across the chromosomes varied from 20 to 80 kb. Population structure analysis grouped the accessions into nine subpopulations. The accessions were evaluated in multi-year, multi-location trials in Olomouc (Czech Republic), Fargo, North Dakota (USA), and Rosthern and Sutherland, Saskatchewan (Canada) from 2013 to 2017. Each trait was phenotyped in at least five location-years. MTAs that were consistent across multiple trials were identified. Chr5LG3_566189651 and Chr5LG3_572899434 for plant height, Chr2LG1_409403647 for lodging resistance, Chr1LG6_57305683 and Chr1LG6_366513463 for grain yield, Chr1LG6_176606388, Chr2LG1_457185, Chr3LG5_234519042 and Chr7LG7_8229439 for seed starch concentration, and Chr3LG5_194530376 for seed protein concentration were identified from different locations and years. This research identified SNP markers associated with important traits in pea that have potential for marker-assisted selection towards rapid cultivar improvement.

## Introduction

Pea (*Pisum sativum* L., 2*n* = 14) is an important cool season pulse crop grown in more than 100 countries on over 12 million hectares worldwide (FAOSTAT 2016; www.fao.org/faostat/en/#data/QC). Pea seeds are considered as a nutritional powerhouse because they are rich in protein, complex carbohydrates, vitamins, minerals and phytochemicals (Burstin et al., 2011). Pea seeds have a large crude protein proportion (∼25% w/w) and high levels of the amino acids lysine and tryptophan, which are relatively low in cereal grains. To enhance the productivity of pea production and meet the global demand for pea consumption, over the last three decades pea breeding programs worldwide have made significant improvement in yield, disease resistance, plant architecture, and lodging resistance (Warkentin et al., 2015). In order to meet future demands, pea breeding must focus both on crop productivity and improving seed quality (Duc et al., 2015).

The use of diverse genetic resources is important for breeding crop varieties (Glaszmann et al., 2010). Crop species with narrow genetic diversity are susceptible to emerging pathogens or other constraints leading to loss of productivity and this may lead to a serious decline in the areas of adaptation (Dyer et al., 2014). Significant morphological diversity exists within pea accessions (Warkentin et al., 2015). The pea leaf type varies from normal with both leaflets and tendrils to semi-leafless that has leaflets replaced by ramified tendrils, and flower color varies from white to reddish-purple (Mikić et al., 2011). Pea growth habit can be indeterminate or determinate, and cotyledon color can be yellow, green or red. Pea accessions also differ substantially in yield potential, ease of harvest, vine length, maturity, seed shape, seed size, and disease resistance (Ouafi et al., 2016; Rana et al., 2017). Thus, knowledge of the genetic diversity of pea accessions is of importance to select genetically diverse parents and to broaden the genetic basis of the cultivated peas.

Initial attempts to estimate the genetic diversity of pea accessions and to assist breeding programs to select diverse accessions were based on a limited number of DNA markers. Tar'an et al. (2005) studied the relations among pea cultivars from USA, Canada, Europe, and Australia using simple sequence repeat (SSR) markers. The cultivars from Canada were observed to group somewhat separately from cultivars from Europe. However, the molecular marker-based genetic similarity did not correlate significantly with similarity based on the agronomic characters, suggesting that the two systems give different estimates of genetic relationship among the varieties. Smýkal et al. (2008) used SSR and retrotransposon-based insertion polymorphism (RBIP) markers to study the genetic diversity of 164 Czech and Slovak pea varieties. The clustering of accessions based on molecular markers did not completely separate the fodder and food types, supporting the findings of Tar'an et al. (2005). Jing et al. (2010) studied the genetic diversity of 3020 *Pisum* accessions using RBIP markers, which separated the landraces, cultivars and wild *Pisum* accessions into distinct groups, and provided a framework for designing core collections. Genetic variation of pea accessions based on SSR markers has also been reported in other studies and the test accessions were clustered into distinct gene pools (Kumari et al., 2013; Jain et al., 2014; Rana et al., 2017; Wu et al., 2017).

Single nucleotide polymorphism (SNP) markers are desirable for estimation of genetic diversity because of their abundance in the genome. SNPs have the ability to discriminate between closely related individuals at a higher resolution. SNP markers have been developed and used to study genetic diversity (Burstin et al., 2015; Diapari et al., 2015; Siol et al., 2017) and genetic mapping in pea (Sindhu et al., 2014; Tayeh et al., 2015a). These genome-wide SNP markers were used to develop SNP arrays for high throughput genotyping of pea germplasm and mapping populations (Sindhu et al., 2014; Tayeh et al., 2015a). Kulaeva et al. (2017) integrated the information of pea gene-based SNP markers from different studies and provided an easy-to-use online tool called the Pea Marker Database. Using next-generation sequencing (NGS) technologies and inexpensive high throughput genotyping platforms, SNPs were used to assess the genetic diversity and to estimate the linkage disequilibrium (LD) in many crop species including pea (Cui et al., 2017; Holdsworth et al., 2017). Using NGS platforms for simultaneous SNP discovery and genotyping, many more SNP markers have been developed and used to construct dense pea linkage maps for the identification of quantitative trait loci (QTLs) for various agronomic and seed quality traits (Tayeh et al., 2015b; Ma et al., 2017; Huang et al., 2017; Gali et al., 2018). While the markers identified in these studies can potentially be used for marker-assisted selection (MAS) of traits in breeding programs, there is also a need to identify additional markers based on a larger gene pool than the bi-parental mapping populations.

Genome-Wide Association Study (GWAS) is an efficient approach to dissect the genetic basis of complex traits using the naturally occurring genetic diversity (Korte and Farlow, 2013). GWAS provides higher mapping resolution than classical bi-parental populations to detect associations between molecular markers and traits of interest, and has been used for identification of markers associated with desirable traits in a wide range of crops (Liu et al., 2016; Cui et al., 2017; Xu et al., 2017). GWAS requires an assessment of the population structure of the diversity panel to determine the genetic relatedness of individuals and minimize detection of false associations (Korte and Farlow, 2013; Sul et al., 2016), and is dependent on the use of an adequately large number of markers. Recent advances in NGS platforms and SNP genotyping provide additional tools to characterize genetic diversity at a high resolution and allow breeders to select for useful diversity to develop new varieties.

The overall objectives of the current study were to characterize the diversity of the genetic sources that are available for pea breeding internationally, and to identify SNP markers associated with agronomic and seed quality traits. A total of 135 accessions from different pea breeding programs around the globe were assembled and used for GWAS. The accessions were genotyped using genotyping-by-sequencing (GBS) method and evaluated in multi-year, multi-location trials for agronomic and seed quality traits.

## Materials and Methods

### Plant Material

The GWAS panel consisted of 135 cultivated pea accessions from 23 breeding programs in Africa (Ethiopia), Asia (India), Australia, Europe (Belarus, Czech Republic, Denmark, France, Lithuania, Netherlands, Russia, Sweden, Ukraine, and United Kingdom), and North America (Canada and USA) as listed in **Table 1**. All the accessions are within the primary gene pool of *Pisum sativum* and most are cultivars released over the past 50 years for local production. The accessions were derived from self-fertilizing lineages, and as such, significant heterozygosity was not expected. All the accessions used were pure lines of F_10_ generation or later, and progeny seeds were used from year to year for phenotyping. All the accessions flowered and matured under the growing conditions at the field test sites, allowing the successful evaluation of the phenotypic traits of interest. The wide distribution of geographic origin and high phenotypic variation of this panel is expected to be a good model to explore the genetic diversity of pea and to identify significant marker-trait associations (MTAs).

**Table 1:** List of pea accessions used as genome-wide association study panel.

Breeding organization/country	Pea accession
Pulse Breeding Australia, Australia	EXCELL(72), KASPA(73), Morgan(71), OZP0805(74), OZP0819(75), OZP0902(76), OZP0903(77), OZP1001(78), OZP1002(79), OZP1004(81), OZP1101(80), OZP1102(84), OZP1104(83), PARAFIELD(85), PBA GUNYAH(86), PBA OURA(87), PBA PERCY(88), PBA TWILIGHT(89) and STURT(90)
Belarus	TMP 15213(142)
Agriculture and Agri-Food Canada, Canada	Agassiz(171), MPG87(141), MP1401(155) and Trapper(165)
Palacký University, Czech Republic	B 99/108(53), Bohatyr(6), Dalibor(48), Dick Trom(49), Hrotovicky Moravska krajova(56), Kamelot(52), Kapucin(59), Klatovsky zeleny(44), Moravsky Hrotovicky krajovy(47), Milion zeleny(45), Moravsky Odeon(51), Prebohatyr(50), Purpurviolett Schottige Nero(57), Slovensky expres(46), Sponsor(54), Stupicka jarni(58) and Terno(55)
Crop Development Centre, University of Saskatchewan, Canada	CDC 1-150-81(169), CDC 1-2347-144(170), CDC Acer(163), CDC Bronco(144), CDC Centennial(145), CDC Dakota(177), CDC Golden(146), CDC Meadow(147), CDC Sage(158), CDC Striker(150), CDC Vienna(167) and Cutlass(143)
McFayden Seed Co., Canada	GRAY'S(36)
Danisco Seeds, Denmark	DS Admiral(148) and Lido(175)
DLF Trifolium, Denmark	Nitouche(152)
Ethiopia	22778(42), 22791(43), G 9173(38), No. 8120(39) and No. 9292(37)
Agriobtentions, France	Dove HR(35)
INRA, Dijon, France	Cameor(135), Carouby de Maussane(60), Champagne(61), Chemin Long(62), Cote D'or(63), D'auvergne(70), Fin de la Bievre(64), Gloire de Correze(65), Merveille D'etampes(66), Normand(67), Picar(68), Piver(69), Serpette Terese(160) and Torsdag(161)
Sarasem, France	Hardy(172) and Cartouche(173)
India	Matar(153) and PLP 105A(41)
Limagrain, Netherlands	Abarth(20), Alfetta(157), Audit(11), Aukland(30), Avantgarde(12), Camry(26), CEB-Montech 4152(28), Cooper(151), Delta(162), Eclipse(149), Emerald(18), Espace(159), Evergreen(19), Garde(25), Lasso(13), Matrix(27), Neon(22), Nette(17), Prophet(24), Quadril(14), Rebel(15), Satelit(16), Sorento(21), Spider(29) and Strada(23)
Lithuania	TMP 15133(137)
Svalof-Weibull, Sweden	Carneval(154) and Highlight(168)
Booker, UK	Radley(166)
John Innes Centre, Norwich, UK	Brutus(132), Enigma-NIAB(134) and Kahuna-NIAB(133)
Russia	AMPLISSIMO ZAZERSKIJ(40), TMP 15159(138), TMP 15202(139) and TMP 15206(140)
Sharpes, UK	Orb(156)
Progene, Othello, WA, USA	Aragorn(176)
Ukraine	Naparnyk(164) and TMP 15116(136)
USDA, Pullman, WA, USA	Lifter(31), Medora(33), Melrose(34), NDP080111(4), NDP080138(5), PS05ND0232(1), PS05ND327(8), PS05ND330(9), PS05ND0434(10), PS07ND0164(2), PS07ND0190(3), Serge(32), Shawnee(7) and Superscout(174)

The number indicated in parenthesis after the name of each accession represents the entry number used for field trials.

### Phenotyping of the GWAS Panel

The GWAS accessions were phenotyped for multiple characteristics in four locations: Sutherland (Canada; 2013–2017), Rosthern (Canada; 2016 and 2017), Fargo, (USA; 2013, 2014, and 2015) and Olomouc (Czech Republic; 2013). In each location and year, the accessions were arranged as a randomized complete block design with two replicates. Plots consisted of 3 rows of 4 m length with 30 cm row spacing and planting density of 75 seeds m^-2^.

The location descriptors are Sutherland (near the city of Saskatoon) (52°12′ N, 106°63' W), Rosthern (52°66′ N, 106°33′ W) in Saskatchewan, Canada, Fargo (47°00′ N, 97°11′ W) in North Dakota, USA, and Olomouc (49°59′ N, 17°25′ E) in Czech Republic. At each location, agronomic practices best suited for field pea production were utilized.

The phenotypes including days to flower, days to maturity, plant height, lodging (1–9 rating scale, 1 = no lodging and 9 = completely lodged (flat) at physiological maturity), grain yield and 1000 seed weight were measured at all locations-years as described by Warkentin et al. (2015). The seeds harvested from selected trials were evaluated for the concentration of acid detergent fiber (ADF), neutral detergent fiber (NDF), starch, and protein, as well as seed shape and seed dimpling according to methods reported by Arganosa et al. (2006) and Ubayasena et al. (2011).

For trait measurements in each trial, normal distribution of residuals and homogeneity of variance were checked using Levene and Shapiro-Wilk tests, respectively (Levene, 1960; Shapiro and Wilk, 1965). Then analysis of variance was conducted for each trait using SAS Proc MIXED (Version 9.4, SAS Institute). The effect of genotype was treated as a fixed factor, while the effect of replication was treated as a random factor. Association of traits among themselves was determined using Pearson correlation coefficients using the correlation function of Mintab18, and significance was declared at P < 0.05.

### Genotyping of the GWAS Panel

The GWAS panel was genotyped using the GBS method following the protocol described by Elshire et al. (2011). For DNA extraction, the GWAS panel was grown in a growth chamber at the University of Saskatchewan phytotron facility. Leaf tissue from a single plant of each accession was harvested and freeze dried. DNA was extracted using the QIAGEN DNeasy 96 plant kit and quantified using picogreen. Individual DNA samples were diluted to 20 ng/µl using 1× TE buffer, pH 8.0.

Two hundred ng of each DNA sample (10 µl volume) was digested with restriction enzymes *Pst*I and *Msp*I, and ligated to unique 4-8 sequence barcode adapters. Five µl aliquots of adapter-ligated DNA samples were pooled in a single tube to produce 59-plex libraries. The pooled DNA was PCR-amplified using sequencing primer followed by purification using a QIAGEN PCR purification kit. For restriction, ligation and PCR amplification, standard experimental conditions as described by Elshire et al. (2011) were followed. The purified DNA library was quantified using a Bioanalyzer (Agilent Technologies) and the 59-plex libraries were sequenced on a single lane of Illumina HiSeq™ 2500 platform (Illumina® Inc., San Diego, CA, USA) using V4 sequencing chemistry at the Sick Kids Hospital, University of Toronto, Canada.

### SNP Variant Calling

The raw reads from Illumina sequencing were assigned to individual accessions based on the 4 to 8 base pair barcode adapters ligated to individual DNA using in-house Perl scripts. Following the deconvolution step, barcode sequences were removed from the read sequences, and the reads were trimmed for quality using the read trimming tool Trimmomatic-0.33. To discover SNP polymorphisms, filtered reads were mapped to the *P. sativum* (cv. Cameor) genome assembly (Kreplak et al., 2019) using the sequence alignment tool Bowtie 2 version 2.2.5. Samtools-1.1 and BCFtools-1.1 were utilized to call variants and saved them in variant call format (VCF). After filtering for a minimum read depth of five and minor allele frequency of 0.05, 16,877 SNP markers were selected and used to determine the population structure and marker-trait association. The selected SNPs were named to represent the corresponding chromosome number, linkage group number, and the base pair position of the SNP.

### Analysis of Population Structure

The population structure of the GWAS panel based on SNP genotyping data was determined by estimating the most likely number of clusters (*K*) into which the accessions could be grouped, and their degree of admixtures, using the program fastSTRUCTURE (Raj et al., 2014). The value of *K* that best fits the data, which is the most likely number of clusters in the population, was determined based on the lowest prediction error, and the smallest number of iterations for convergence. From the matrix of contributions, *Q* the probabilities of belonging to one of the clusters were derived, and accessions assigned accordingly. An unweighted neighbor-joining (NJ) tree was constructed using a shared allele index based on a dissimilarity matrix estimated from the SNP dataset (Perrier et al., 2003).

### Linkage Disequilibrium Analysis

LD of SNP markers of each chromosome was calculated as the correlation between marker-pairs calculated as Pearson correlation coefficient (r). LD decay was calculated by Quantile regression (R package 'quantreg'; Koenker 2017) by plotting r^2^ values as a function of genetic distance.

### Association Analysis

The association between SNP genotypes and the phenotypes was determined using the software GAPIT (Genome Association and Prediction Integrated Tool – R package; Lipka et al., 2012). The Q values, which consider the genetic structure of the GWAS panel, and the kinship coefficient matrix (K) that explains the most probable identity by state of each allele between accessions, were used in the analysis. Mixed linear method (MLM) and SUPER (Tang et al., 2016) were tested for association analysis. MLM was run using K values calculated by GAPIT and identity-by-state (IBS) methods, and principal co-ordinate values as covariates. To select the appropriate model for association analysis, the quantile-quantile (Q-Q) plots of each drawn between the observed and expected log_10_ P values were compared, and the MLM based on Q and K values from IBS was used for association analysis.

## Results

### Genotyping of the GWAS Panel

**Figure 1:**
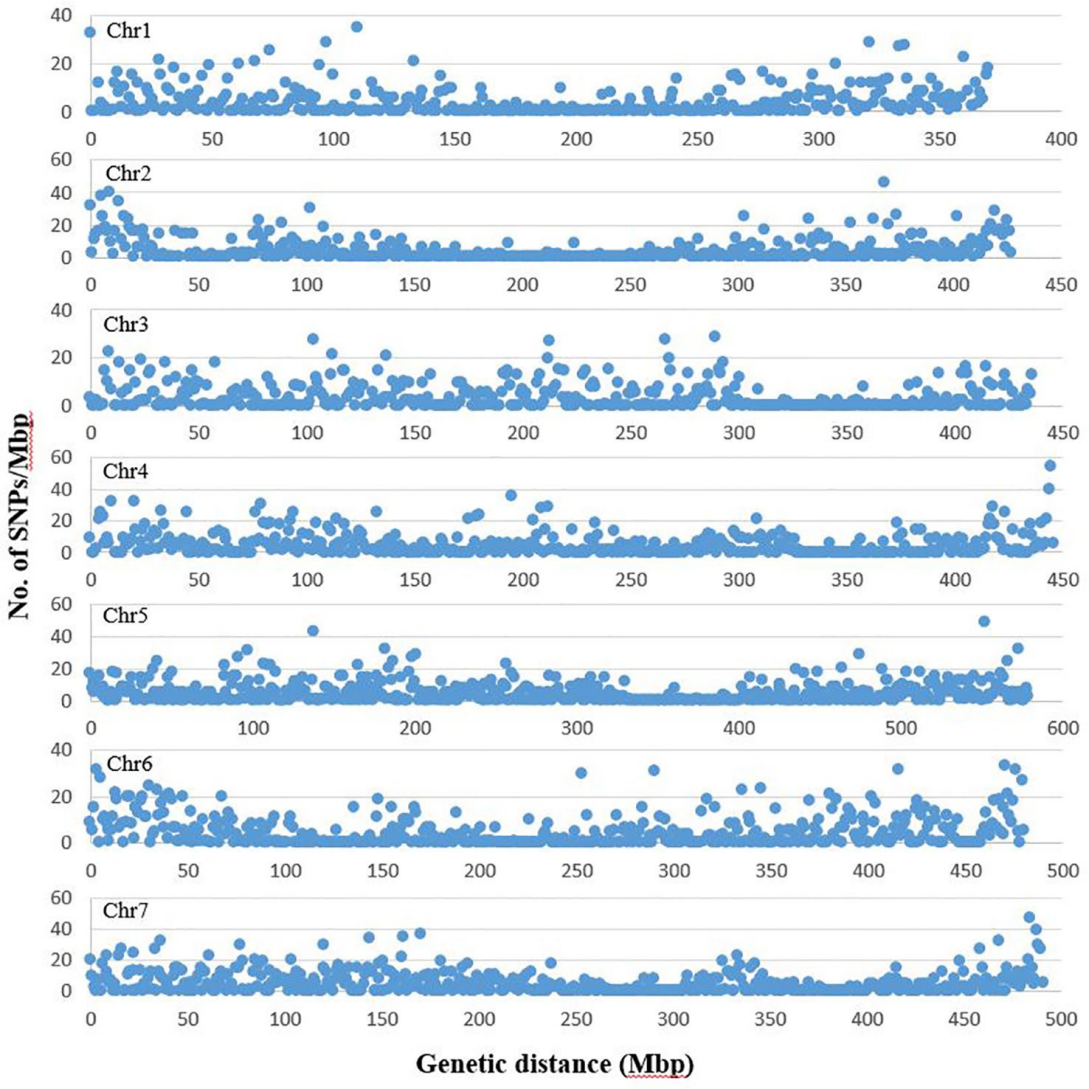
Distribution of SNP markers selected for population structure and trait association analysis across the seven chromosomes of pea. The graph represents number of SNPs in each million bp of genetic distance of the seven pea chromosomes. The chromosome and linkage group assignment was in accordance to the pea genome assembled by Kreplak et al. (2019). The graphs are based on number of SNPs identified on chromosomes 1 to 7 (1685, 1768, 1786, 2356, 2917, 2349 and 2747, respectively).

From the three lanes of sequencing on HiSeq™ 2500, a total of 1005.1 million reads of 100 bp length were obtained with a minimum of 1.47 million and maximum of 12.9 million reads per accession. The average Q30 ratio and guanine–cytosine (GC) content of the reads were 92.3 and 44.1%, respectively. Of the raw reads, 98.0% remained after trimming for barcode adapter sequences and quality. These high quality reads were aligned to the pea genomic sequence (Kreplak et al., 2019). On average, 60.5% of the reads per accession were aligned to the reference sequence and 91.9% of the aligned sequences were uniquely aligned. After filtering the identified SNPs for a minimum allele frequency (MAF) of 0.05 and minimum read depth of five, 16,877 SNPs were selected and used for analysis of population structure and marker-trait association. Of the selected SNPs, 15,608 loci were located on the seven chromosomes of pea (**Figure 1**). The remaining 1269 markers were chromosomally non-assigned, and were designated by their corresponding scaffolds or superscaffolds. The SNP markers were named according to their assigned chromosome and linkage group followed by the base pair position within the chromosome. The designation of chromosomes and linkage groups is in accordance with the pea genome sequence assembled by Kreplak et al. (2019).

### Linkage Disequilibrium Analysis

**Figure 2:**
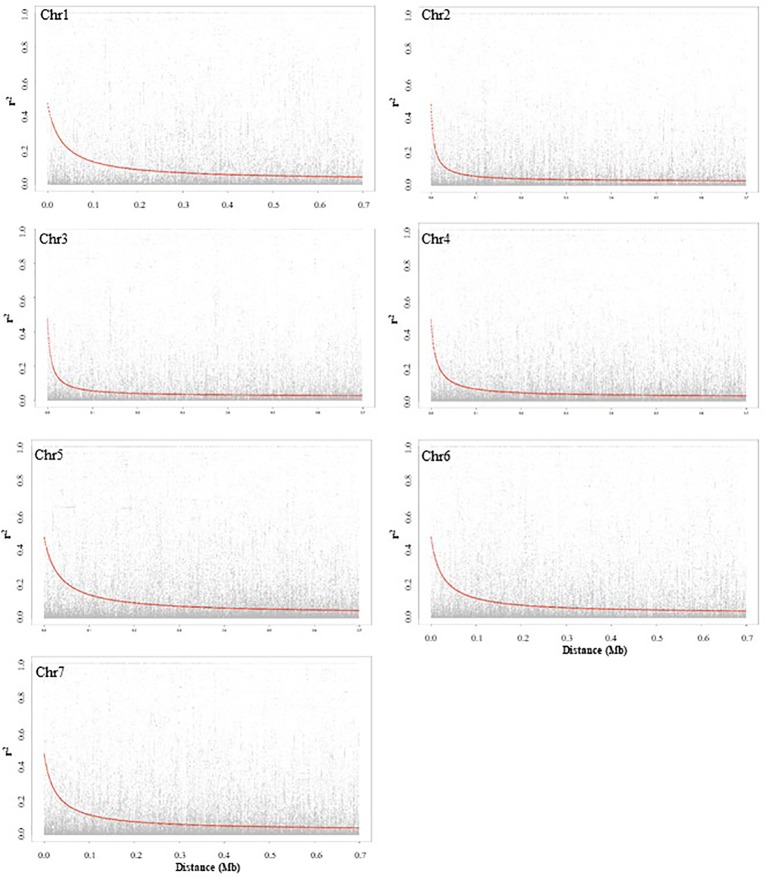
Chromosome-wise linkage disequilibrium decay based on 135 pea accessions. The decline of LD- r^2^ between SNPs pairs is presented as a function of physical distance in base pairs.

LD decay based on SNP markers of each chromosome was calculated as the Pearson correlation coefficient (r^2^) between marker pairs. The r^2^_max,90_, which is the maximum r^2^ achieved in the 90th percentile of chromosomes 1 to 7 is 0.35, 0.25, 0.26, 0.24, 0.32, 0.32, and 0.29, respectively. The LD decay varied among the seven chromosomes, and chromosomes 2 and 5 had the most rapid and slowest decay, respectively. The LD_1/2max,90_ of chromosomes 1 to 7, which is the physical distance in Mb at which LD has decayed to half of r^2^_max,90_ is 0.06, 0.02, 0.02, 0.04, 0.08, 0.06, and 0.07, respectively. LD plots of each chromosome are presented in **Figure 2**.

### Genetic Structure of GWAS Accessions

**Figure 3:**
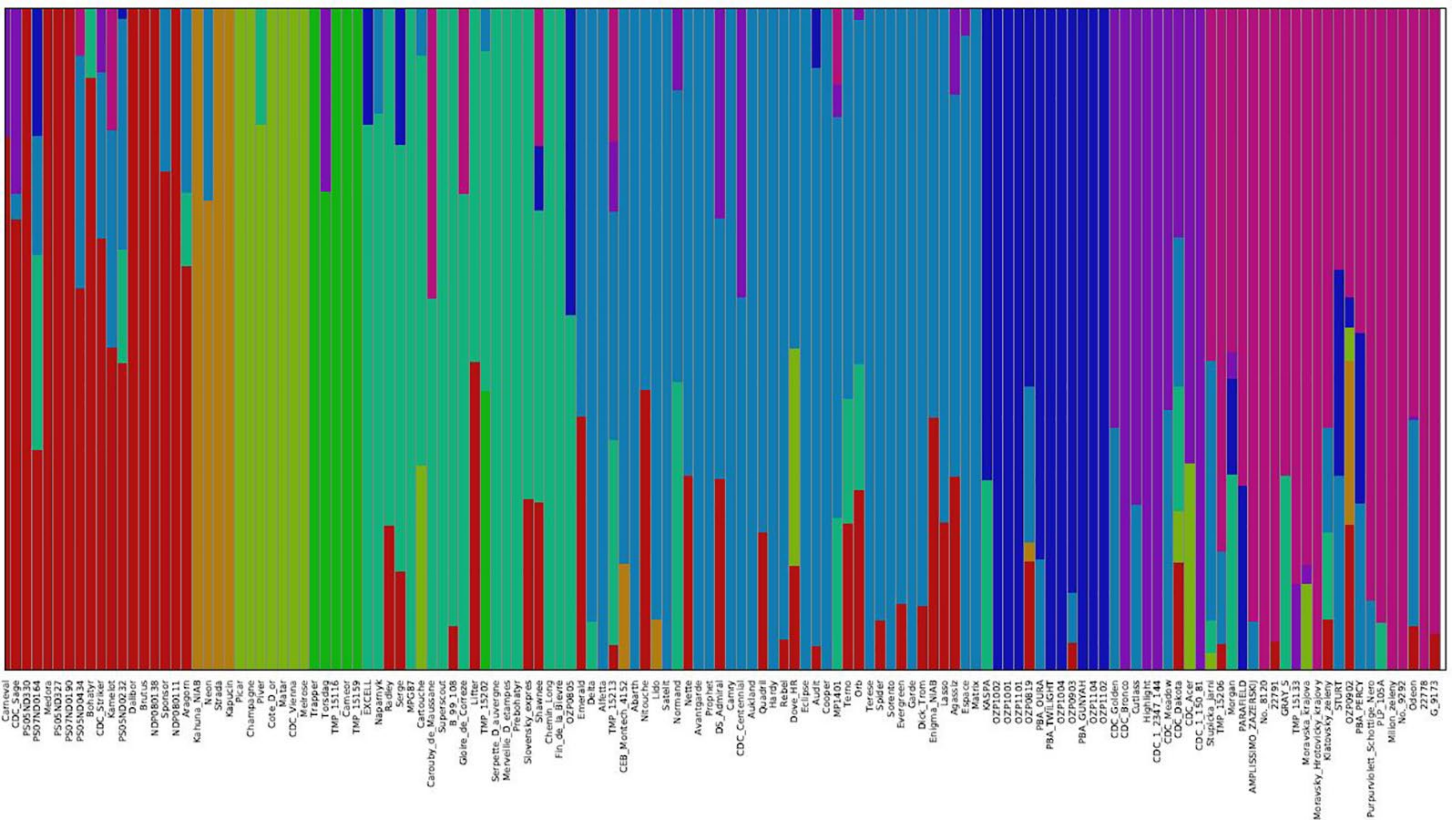
Population structure of 135 pea accessions based on *K* = 9. In the panel, each accession is indicated as a vertical bar partitioned into colored segments where the respective length of these segments represents the proportion of the individual's genome in a given group.

The genetic structure of the 135 accessions was analyzed using fastSTRUCTURE. Model-based, maximum likelihood ancestry estimation procedure was used for the analysis. The most likely number of clusters (k) was tested from 2 to 10, and a k-value of 9 was selected to describe the genetic structure of the 135 accessions. The admixture analysis estimated the probability of membership of each individual accession to each cluster (**Figure 3**). The corresponding Q-matrix at k = 9 was used for marker-trait association analysis. The admixture analysis assigned individual accessions to clusters to study hybrid regions of the genome, and identified common ancestry of accessions from different pea breeding programs. In general, accessions from specific breeding programs tended to cluster together.

In cluster 1, 10 accessions from USA breeding programs clustered with 2 accessions from Canada, 4 accessions from Czech Republic, Carneval from Sweden, and Brutus from United Kingdom, and showed varying degrees of hybrid zones from accessions of other geographical regions. The four accessions, Kahuna (John Innes Centre, UK), Neon (Limagrain, Netherlands), Strada (Limagrain, Netherlands), and Kapucin (Palacky Univeristy, Czech Republic), which formed cluster 2 are accessions of marrowfat market class characterized by large green cotyledon seeds with blocky seed shape used typically as snack foods. Seven accessions from four breeding programs formed cluster 3, and six of the accessions had no admixture from other clusters. Some of these accessions Champagne (INRA, France), CDC Vienna (CDC, Canada) and Melrose (USDA, USA) are known to have greater frost tolerance. Five older pea accessions from different breeding programs formed cluster 4, of which Trapper and Torsdag are known forage pea accessions. Clusters 5 and 6 are comprised of 20 and 38 accessions from multiple breeding programs, respectively. The accessions in cluster 5 are relatively older varieties and cluster 6 has many relatively recent western European varieties (like Delta, Alfetta, Nitouche, Lido) and a few Canadian varieties (like Agassiz, MP1401 and CDC Centennial). Twelve of the 19 accessions from Pulse Breeding Australia (PBA) clustered together in cluster 7. Eight of the 12 accessions from CDC, Canada and Highlight from Svalof-Weibull (Sweden) formed cluster 8. The four accessions in this cluster which had no admixture are CDC Bronco, Highlight (parent of CDC Bronco), CDC 1-150-81, and CDC 1-2347-144 (the two latter are mutants of CDC Bronco). Cluster 9 has many accessions from Eastern European programs and all five accessions from Ethiopia.

**Figure 4:**
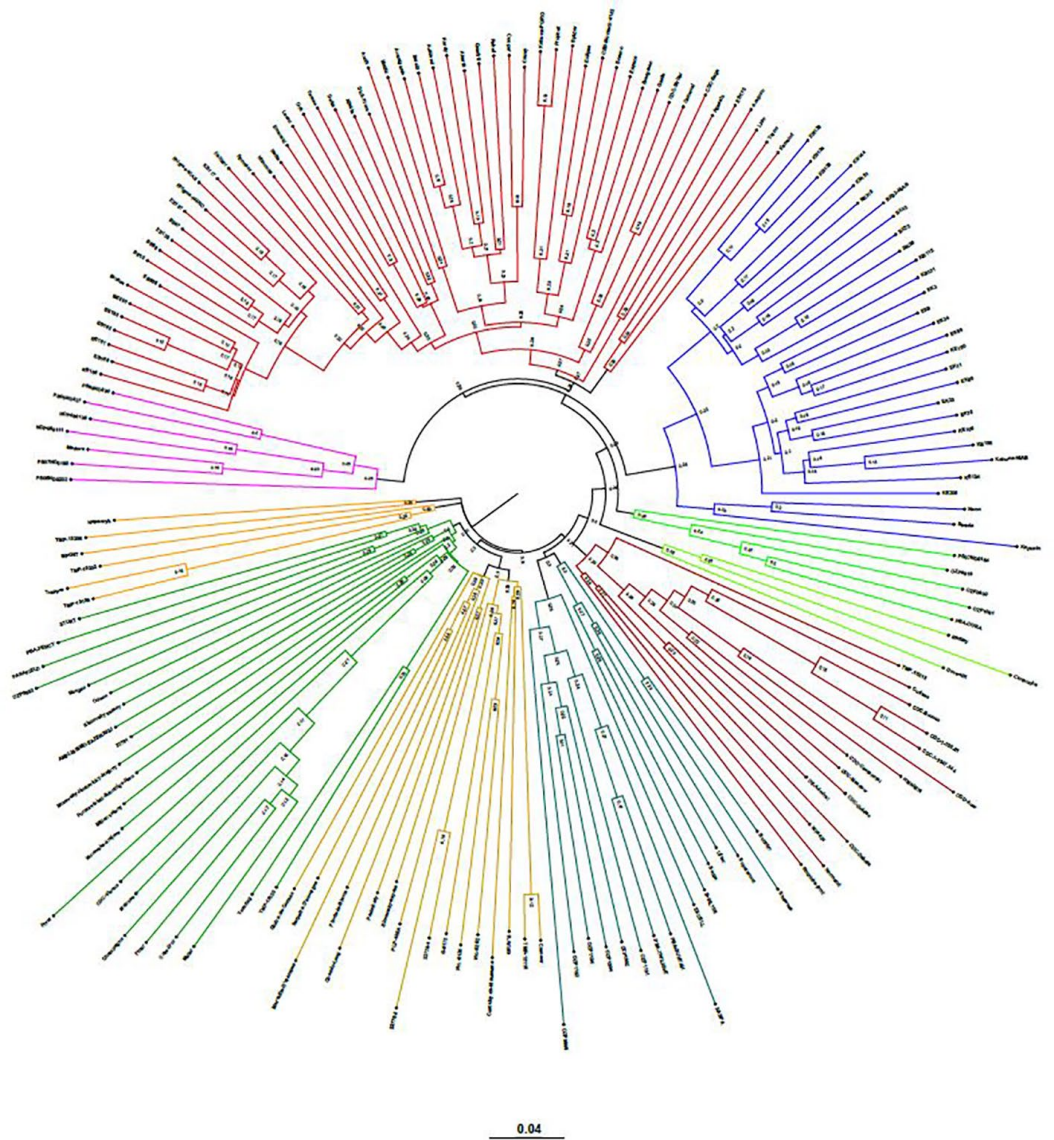
Genetic relatedness among the 135 pea accessions estimated by neighbor-joining method and represented as a polar tree diagram. The estimated genetic relatedness is based on 16,877 SNPs identified by genotyping-by-sequencing and filtered for minor allele frequency of 0.05.

The neighbor-joining (NJ) tree presented in **Figure 4** is based on the shared-allele genetic distance. The grouping of phylogenetic clusters differed to some extent from the grouping of accessions based on the extent of admixture as shown in **Figure 3**. For example, the 18 accessions represented as cluster 1 in structure analysis, were regrouped with 9 accessions as one cluster, 7 accessions as another cluster along with other accessions. Two accessions PS07ND0164 and Bohatyr of cluster 1 and four accessions Kahuna-NIAB, Neon, Strada and Kapucin of cluster 2 in structure analysis were grouped as one phylogenetic cluster along with accessions from Australia. In structure analysis, 12 accessions from PBA formed cluster 7, while accessions EXCELL and OZP0805 from PBA were grouped in cluster 5. In the NJ tree, these fourteen accessions from PBA were clustered together along with accessions from other sources. The nine accessions in cluster 8 of the admixture plot (**Figure 3**), along with DS-Admiral and CDC Centennial which showed significant admixture from this cluster, were part of one cluster in the NJ tree.

### Phenotypic Measurements

Phenotypic data collected for the GWAS panel in multi-location, multi-year trials are summarized in **Table 2**. The accessions varied widely in the characteristics measured. The days to flowering (DTF) varied significantly within the GWAS panel by an average of 16.8 days between the early flowering and late flowering accessions compared across the years and locations. In comparison the accessions differed by 18.1 days in days to maturity (DTM). Substantial variation of plant height was observed, where the average of minimum and maximum plant height measured across the trials is 43.7 and 151.3 cm, respectively. In terms of lodging resistance, the accessions varied from a score of 1.0 to 9.0 measured on a 1-9 rating scale. The yield of individual accessions ranged from less than 100 kg/ha to >6000 kg/ha. The seed weight of the accessions, measured as 1000 seed weight, varied from 70 g to 436 g. The GWAS accessions were also quite diverse for seed dimpling and seed shape.

**Table 2:** Minimum, maximum and mean values of phenotypic traits measured in 135 pea accessions of genome-wide association study panel.

Trait	Station/year	Minimum	Maximum	Mean	Standard Deviation
Days to flowering	2013 Fargo	29.3	58.0	39.9	0.8
	2013 Olomouc	50.0	64.0	56.0	2.5
	2013 Sutherland	46.0	57.0	52.1	0.6
	2014 Fargo	39.0	54.0	45.2	1.0
	2014 Sutherland	54.0	64.5	58.4	0.4
	2015 Fargo	37.0	54.0	45.0	0.5
	2015 Sutherland	57.0	73.0	67.6	0.6
	2016 Rosthern	45.5	62.0	55.9	0.7
	2016 Sutherland	50.0	67.0	59.6	0.9
	2017 Rosthern	43.5	59.0	53.5	0.5
	2017 Sutherland	44.0	67.0	60.7	0.8
	Average	45.0	61.8	54.0	0.8
Days to maturity	2013 Fargo	70.0	97.0	85.0	1.9
	2013 Olomouc	75.0	87.5	82.0	2.4
	2013 Sutherland	90.0	101.0	95.1	1.2
	2014 Fargo	74.5	97.5	85.0	1.8
	2014 Sutherland	82.0	101.5	94.6	0.9
	2015 Fargo	76.5	97.0	89.2	1.7
	2015 Sutherland	93.0	110.5	100.2	1.6
	2016 Rosthern	89.0	105.0	97.6	1.6
	2016 Sutherland	88.5	105.5	97.7	1.9
	2017 Rosthern	82.5	97.0	90.0	1.9
	2017 Sutherland	85.0	105.5	94.7	1.6
	Average	82.4	100.5	91.9	1.7
Plant height (cm)	2013 Fargo	52.3	185.5	96.7	6.5
	2013 Olomouc	14.5	90.5	49.8	6.0
	2013 Sutherland	55.5	180.0	84.9	19.7
	2014 Sutherland	35.3	156.2	87.2	8.2
	2015 Sutherland	40.5	130.7	70.8	5.6
	2016 Rosthern	53.8	163.8	91.6	5.8
	2016 Sutherland	52.2	175.0	101.7	8.2
	2017 Rosthern	50.2	127.2	87.3	5.1
	2017 Sutherland	39.0	153.2	88.0	6.9
	Average	43.7	151.3	84.2	8.0
Lodging resistance (1-9)	2013 Fargo	1.0	7.8	3.3	0.3
	2013 Olomouc	4.0	9.0	6.7	0.9
	2013 Sutherland	3.5	9.0	6.2	0.5
	2014 Fargo	2.5	9.0	6.9	0.8
	2014 Sutherland	2.0	9.0	5.9	0.4
	2015 Sutherland	2.0	9.0	5.9	0.5
	2016 Rosthern	2.0	8.5	5.5	0.6
	2016 Sutherland	2.0	9.0	5.5	0.6
	2015 Fargo	2.0	9.0	6.1	0.6
	2017 Sutherland	2.0	9.0	5.3	0.5
	Average	2.3	8.8	5.7	0.6
Yield (kg/ha)	2013 Fargo	31	4835	2621	497
	2013 Sutherland	1229	4125	2744	211
	2014 Fargo	55	2821	1504	209
	2014 Sutherland	160	3954	2148	451
	2015 Fargo	324	5849	3376	427
	2015 Sutherland	1047	3824	2450	223
	2016 Rosthern	929	6858	4399	355
	2016 Sutherland	1606	4800	3413	456
	2017 Rosthern	1787	6078	4166	420
	2017 Sutherland	1836	5382	3295	330
	Average	901	4853	3011	358
1000 seed weight (g)	2013 Fargo	70	294	171	16
	2014 Fargo	85	442	209	8
	2015 Fargo	81	411	185	9
	2016 Rosthern	78	430	224	7
	2016 Sutherland	89	383	196	10
	2017 Rosthern	92	348	222	6
	2017 Sutherland	106	436	225	4
	Average	86	392	204	9
Seed dimpling (%)	2015 Fargo	5	78	31	16
	2015 Sutherland	8	100	45	24
	2016 Rosthern	0	100	33	7
	2016 Sutherland	0	100	45	8
	2017 Rosthern	0	100	6	2
	2017 Sutherland	0	100	26	7
	Average	2	96	31	11
Seed shape (1-5 scale)	2015 Fargo	2.0	4.8	3.1	0.5
	2015 Sutherland	2.3	5.0	3.0	0.4
	2016 Rosthern	1.5	5.0	3.0	0.1
	2016 Sutherland	1.5	5.0	3.0	0.1
	2017 Rosthern	1.0	5.0	3.0	0.2
	2017 Sutherland	1.0	5.0	3.2	0.2
	Average	1.5	5.0	3.1	0.3
Acid detergent fiber (%)	2013 Sutherland	3.2	8.4	5.9	0.2
	2016 Rosthern	7.9	14.9	10.1	0.5
	2016 Sutherland	7.3	14.3	10.1	0.6
	2017 Rosthern	8.0	15.9	10.3	0.4
	2017 Sutherland	7.1	14.2	9.6	0.5
	Average	6.7	13.5	9.2	0.5
Neutral detergent fiber (%)	2013 Sutherland	7.4	17.7	11.9	0.4
	2016 Rosthern	13.6	25.6	16.8	0.8
	2016 Sutherland	14.0	23.8	16.9	0.7
	2017 Rosthern	12.9	26.3	15.8	0.6
	2017 Sutherland	12.1	21.9	15.2	0.6
	Average	12.0	23.1	15.3	0.6
Seed starch (%)	2013 Sutherland	17.8	44.0	38.7	1.0
	2016 Rosthern	33.8	54.7	49.2	1.4
	2016 Sutherland	38.8	58.3	52.2	1.3
	2017 Rosthern	28.2	56.8	49.2	1.2
	2017 Sutherland	37.9	55.0	50.1	1.0
	Average	31.3	53.8	47.9	1.2
Seed crude protein (%)	2013 Sutherland	19.1	28.3	22.8	0.9
	2016 Rosthern	22.1	30.2	25.8	0.8
	2016 Sutherland	18.7	29.3	23.4	0.9
	2017 Rosthern	20.6	30.9	24.3	0.9
	2017 Sutherland	20.6	28.4	23.6	0.8
	Average	20.2	29.4	24.0	0.9

The GWAS panel is also highly diverse for the seed quality traits measured as percentage of acid detergent fiber, neutral detergent fiber, starch, and protein content. The acid and neutral detergent fiber concentrations varied from 3.2% and 7.4% to 15.9% and 26.3%, respectively. The starch concentration varied from 17.8% to 58.3%, and protein concentration varied from 19.1% to 30.9%. Overall, there is sufficient phenotypic diversity in the GWAS panel, in terms of agronomic traits, seed morphology and seed quality traits, to support association analysis.

### Association Analysis

Of the MTAs identified for individual trials, 251 MTAs as listed in **Table 3** were selected based on their P value and occurrence in multiple trials. The flanking sequences of the markers listed were provided in **Table S1**. Nine markers, positioned on chromosomes 1, 2, 4 and 6, and three non-chromosomal scaffolds were associated with DTF in at least four trials, and on average each marker explained 3-11% of the phenotypic variance (PV) measured as the difference in R-square of the model with the SNP and without the SNP. SNP marker Chr1LG6_362652367 was associated with DTF in seven of the 11 trials. Five markers, four on chromosome 3 and one on chromosome 5 were associated with DTM in multiple trails. SNP marker Chr3LG5_126657675 was associated with DTM in eight of the 11 trials.

**Table 3:** Trait linked SNP markers identified by association analysis of various pea phenotypes using the mixed linear model (MLM).

**Trait**	**SNP**	**Year/Station**	**P value**	**R-square of model with SNP**	**R-square of marker^†^**	**Average R-square of marker^†^**
Days to Flowering (11)	Chr1LG6_362652367	2014 Fargo	1.08E-03	0.50	0.05	
		2015 Fargo	1.17E-03	0.44	0.06	
		2016 Rosthern	1.34E-03	0.29	0.07	
		2017 Rosthern	1.36E-04	0.45	0.08	
		2014 Sutherland	9.47E-04	0.38	0.07	
		2016 Sutherland	1.79E-03	0.34	0.06	
		2017 Sutherland	2.79E-03	0.30	0.06	0.07
	Chr1LG6_366513463	2013 Fargo	6.95E-04	0.39	0.07	
		2015 Fargo	1.02E-03	0.44	0.06	
		2017 Rosthern	5.38E-03	0.41	0.04	
		2015 Sutherland	4.50E-04	0.34	0.08	
		2016 Sutherland	6.77E-04	0.36	0.07	
		2017 Sutherland	1.66E-03	0.31	0.07	0.07
	Chr2LG1_374429941	2016 Rosthern	5.92E-04	0.30	0.08	
		2015 Sutherland	9.42E-04	0.33	0.07	
		2016 Sutherland	2.23E-03	0.34	0.06	
		2017 Sutherland	1.73E-03	0.31	0.07	0.07
	Chr4LG4_223948832	2013 Fargo	9.90E-04	0.39	0.07	
		2014 Fargo	8.16E-04	0.50	0.06	
		2015 Fargo	3.07E-04	0.46	0.07	
		2017 Rosthern	1.21E-03	0.43	0.06	
		2014 Sutherland	3.10E-04	0.40	0.08	
		2016 Sutherland	4.22E-03	0.33	0.05	0.06
	Chr4LG4_255086751	2014 Fargo	1.87E-03	0.49	0.05	
		2015 Fargo	3.94E-04	0.45	0.07	
		2017 Rosthern	2.98E-03	0.42	0.05	
		2014 Sutherland	5.28E-04	0.39	0.07	
		2016 Sutherland	2.14E-03	0.34	0.06	0.06
	Chr6LG2_159951043	2016 Rosthern	8.14E-04	0.30	0.08	
		2017 Rosthern	2.36E-04	0.45	0.08	
		2013 Sutherland	1.23E-03	0.31	0.07	
		2016 Sutherland	1.39E-04	0.38	0.10	
		2017 Sutherland	1.06E-03	0.31	0.07	0.08
	Sc00936_29805	2013 Fargo	1.30E-03	0.39	0.06	
		2016 Rosthern	3.25E-04	0.31	0.09	
		2017 Rosthern	1.22E-03	0.43	0.06	
		2013 Sutherland	6.88E-04	0.32	0.08	
		2015 Sutherland	7.57E-04	0.34	0.08	
		2016 Sutherland	1.11E-03	0.35	0.07	
		2017 Sutherland	3.85E-04	0.33	0.09	0.08
	Sc01142_238	2015 Fargo	1.77E-03	0.44	0.05	
		2016 Rosthern	8.74E-04	0.30	0.08	
		2017 Rosthern	1.03E-03	0.43	0.06	
		2016 Sutherland	3.00E-03	0.34	0.06	
		2017 Sutherland	9.87E-04	0.32	0.07	0.06
	Sc03817_83023	2014 Fargo	3.80E-04	0.51	0.06	
		2015 Fargo	1.25E-04	0.47	0.08	
		2017 Rosthern	6.54E-04	0.44	0.07	
		2014 Sutherland	2.46E-05	0.43	0.11	
		2015 Sutherland	1.66E-03	0.33	0.07	
		2016 Sutherland	3.88E-04	0.36	0.08	
		2017 Sutherland	2.85E-03	0.30	0.06	0.08
Days to Maturity (11)	Chr3LG5_106358046	2015 Fargo	5.29E-04	0.39	0.08	
		2017 Rosthern	1.89E-03	0.46	0.05	
		2017 Sutherland	5.69E-04	0.42	0.07	0.07
	Chr3LG5_112288560	2013 Fargo	4.47E-03	0.32	0.06	
		2014 Fargo	1.55E-05	0.65	0.08	
		2017 Rosthern	1.69E-03	0.46	0.05	
		2014 Sutherland	4.14E-04	0.51	0.06	
		2016 Sutherland	6.75E-04	0.46	0.06	
		2017 Sutherland	3.28E-04	0.42	0.08	0.07
	Chr3LG5_112351959	2014 Fargo	5.05E-06	0.66	0.09	
		2017 Rosthern	1.27E-03	0.46	0.06	
		2014 Sutherland	4.72E-04	0.51	0.06	
		2016 Sutherland	1.08E-03	0.46	0.06	
		2017 Sutherland	4.34E-04	0.42	0.08	0.07
	Chr3LG5_126657675	2013 Fargo	8.35E-04	0.35	0.08	
		2014 Fargo	1.67E-05	0.65	0.08	
		2017 Rosthern	6.76E-03	0.45	0.04	
		2013 Sutherland	3.55E-04	0.55	0.06	
		2014 Sutherland	4.30E-05	0.53	0.09	
		2015 Sutherland	1.74E-03	0.70	0.03	
		2016 Sutherland	1.98E-03	0.45	0.05	
		2017 Sutherland	2.29E-04	0.43	0.08	0.06
	Chr5LG3_253287072	2013 Fargo	2.35E-03	0.33	0.06	
		2015 Fargo	3.41E-03	0.37	0.05	
		2015 Sutherland	4.12E-04	0.71	0.04	
		2017 Sutherland	7.23E-04	0.42	0.07	0.06
Plant Height (9)	Chr5LG3_566189651	2013 Fargo	2.10E-04	0.47	0.08	
		2016 Rosthern	4.82E-06	0.67	0.08	
		2017 Rosthern	1.33E-05	0.48	0.11	
		2014 Sutherland	3.29E-07	0.70	0.11	
		2015 Sutherland	2.65E-06	0.71	0.08	
		2016 Sutherland	9.72E-06	0.62	0.09	
		2017 Sutherland	1.52E-06	0.67	0.10	0.09
	Chr5LG3_572899434	2013 Fargo	5.09E-04	0.46	0.07	
		2016 Rosthern	1.38E-05	0.66	0.07	
		2017 Rosthern	1.10E-04	0.46	0.09	
		2014 Sutherland	6.09E-06	0.68	0.08	
		2015 Sutherland	2.77E-06	0.71	0.08	
		2016 Sutherland	6.71E-07	0.64	0.12	
		2017 Sutherland	2.60E-06	0.66	0.09	0.09
	Chr5LG3_573518168	2016 Rosthern	7.19E-05	0.64	0.06	
		2017 Rosthern	2.90E-05	0.47	0.10	
		2014 Sutherland	1.89E-05	0.67	0.07	
		2015 Sutherland	7.20E-06	0.71	0.07	
		2016 Sutherland	2.37E-05	0.61	0.08	
		2017 Sutherland	1.58E-06	0.67	0.10	0.08
	Chr5LG3_573697426	2016 Rosthern	2.43E-05	0.65	0.07	
		2017 Rosthern	2.18E-05	0.47	0.11	
		2015 Sutherland	1.14E-06	0.72	0.08	
		2017 Sutherland	3.63E-06	0.66	0.09	0.09
Lodging resistance (10)	Chr1LG6_323387498	2015 Fargo	9.23E-04	0.60	0.04	
		2013 Sutherland	2.02E-03	0.66	0.03	
		2016 Sutherland	6.60E-05	0.77	0.04	0.04
	Chr2LG1_47522665	2015 Sutherland	1.84E-03	0.75	0.02	
		2016 Sutherland	3.55E-04	0.76	0.03	
		2017 Sutherland	4.97E-05	0.74	0.05	0.03
	Chr2LG1_409403647	2013 Sutherland	5.13E-05	0.69	0.06	
		2014 Sutherland	1.63E-04	0.71	0.04	
		2016 Sutherland	1.80E-04	0.77	0.03	
		2017 Sutherland	2.52E-04	0.73	0.04	0.04
	Chr3LG5_415353144	2013 Sutherland	1.42E-03	0.67	0.04	
		2014 Sutherland	2.26E-04	0.71	0.04	
		2016 Sutherland	9.90E-04	0.76	0.03	0.03
	Chr5LG3_192474110	2013 Sutherland	2.67E-03	0.66	0.03	
		2014 Sutherland	6.52E-04	0.70	0.04	
		2015 Sutherland	4.06E-03	0.74	0.02	0.03
						
Yield (10)	Chr1LG6_57305683	2014 Fargo	1.69E-03	0.69	0.03	
		2016 Rosthern	1.76E-03	0.55	0.04	
		2014 Sutherland	1.90E-03	0.38	0.06	0.05
	Chr1LG6_366513463	2016 Rosthern	9.70E-04	0.56	0.05	
		2015 Sutherland	3.30E-03	0.52	0.04	
		2017 Sutherland	2.96E-03	0.47	0.05	0.05
	Chr4LG4_373933955	2016 Rosthern	6.12E-04	0.56	0.05	
		2017 Sutherland	1.60E-03	0.47	0.05	0.05
	Chr7LG7_488770913	2014 Fargo	4.56E-04	0.70	0.04	
		2017 Rosthern	2.36E-04	0.67	0.05	0.04
	SSc4454_324798	2013 Fargo	6.41E-04	0.57	0.06	
		2015 Fargo	2.51E-03	0.78	0.02	0.04
Seed weight (7)	Chr1LG6_176606388	2014 Fargo	7.68E-04	0.77	0.03	
		2015 Fargo	2.59E-04	0.79	0.03	
		2017 Rosthern	3.51E-04	0.76	0.03	
		2016 Sutherland	7.12E-04	0.73	0.03	0.03
	Sc00398_17041	2013 Fargo	4.49E-04	0.60	0.05	
		2014 Fargo	3.61E-04	0.77	0.03	
		2015 Fargo	8.17E-05	0.80	0.04	
		2016 Rosthern	1.40E-04	0.77	0.04	
		2017 Rosthern	2.27E-04	0.76	0.03	
		2016 Sutherland	5.70E-04	0.73	0.03	0.04
	Sc01126_54371	2013 Fargo	4.01E-04	0.60	0.05	
		2014 Fargo	3.24E-04	0.77	0.03	
		2015 Fargo	6.48E-05	0.80	0.04	
		2016 Rosthern	5.33E-04	0.76	0.03	
		2017 Rosthern	4.50E-04	0.76	0.03	
		2016 Sutherland	8.77E-04	0.73	0.03	0.03
	Sc01886_124838	2014 Fargo	1.02E-04	0.78	0.04	
		2015 Fargo	3.89E-05	0.80	0.04	
		2016 Rosthern	2.12E-05	0.78	0.05	
		2017 Rosthern	2.60E-04	0.76	0.03	
		2016 Sutherland	4.79E-05	0.75	0.05	0.04
Seed Shape (6)	Chr3LG5_197482300	2016 Rosthern	3.23E-05	0.41	0.11	
		2016 Sutherland	1.27E-04	0.41	0.09	
		2017 Sutherland	1.59E-04	0.42	0.09	0.10
	Chr6LG2_68264764	2016 Rosthern	1.22E-04	0.40	0.09	
		2017 Rosthern	4.46E-05	0.40	0.11	
		2016 Sutherland	1.19E-04	0.41	0.09	
		2017 Sutherland	5.67E-05	0.43	0.10	0.10
	Chr6LG2_372463590	2016 Rosthern	2.76E-04	0.39	0.08	
		2017 Rosthern	7.05E-04	0.37	0.07	
		2016 Sutherland	1.07E-04	0.42	0.09	0.08
	Chr7LG7_7724682	2016 Rosthern	6.84E-05	0.40	0.10	
		2017 Rosthern	4.84E-04	0.37	0.08	
		2016 Sutherland	2.58E-05	0.43	0.11	
		2017 Sutherland	3.48E-04	0.41	0.08	0.09
Seed dimpling (6)	Chr1LG6_46289124	2017 Sutherland	6.92E-04	0.40	0.07	
		2017 Rosthern	7.07E-04	0.46	0.06	
		2016 Rosthern	3.39E-03	0.38	0.05	0.06
	Chr1LG6_100615820	2016 Sutherland	2.19E-04	0.54	0.07	
		2017 Sutherland	6.11E-04	0.40	0.07	
		2017 Rosthern	8.91E-04	0.45	0.06	
		2016 Rosthern	2.30E-03	0.39	0.06	0.06
	Chr3LG5_13738628	2016 Rosthern	2.70E-04	0.41	0.08	
		2017 Sutherland	7.46E-04	0.40	0.07	0.08
Acid detergent fiber (5)	Chr5LG3_301190005	2017 Sutherland	4.54E-04	0.49	0.06	
		2017 Rosthern	5.03E-04	0.45	0.07	
		2016 Sutherland	1.49E-03	0.45	0.05	0.06
	Chr6LG2_372463590	2017 Sutherland	5.32E-04	0.49	0.06	
		2017 Rosthern	5.40E-04	0.45	0.07	
		2013 Sutherland	6.13E-04	0.46	0.06	0.06
	Chr6LG2_68264764	2013 Sutherland	2.13E-07	0.55	0.16	
		2016 Rosthern	3.03E-04	0.49	0.07	
		2017 Sutherland	9.09E-04	0.49	0.06	
		2016 Sutherland	1.40E-03	0.45	0.06	0.08
	Chr7LG7_7724682	2016 Sutherland	7.00E-05	0.49	0.09	
		2017 Rosthern	2.61E-04	0.46	0.07	
		2017 Sutherland	3.67E-04	0.50	0.07	0.08
	Sc03839_38033	2016 Sutherland	1.84E-04	0.47	0.08	
		2013 Sutherland	2.56E-04	0.47	0.07	
		2017 Rosthern	3.11E-04	0.46	0.07	0.07
Neutral detergent fiber (5)	Chr2LG1_457185	2016 Rosthern	2.23E-04	0.48	0.07	
		2017 Rosthern	2.16E-04	0.42	0.08	
		2017 Sutherland	2.17E-04	0.43	0.08	0.08
	Chr3LG5_64217010	2016 Sutherland	5.93E-04	0.34	0.08	
		2017 Sutherland	3.95E-05	0.45	0.10	0.09
	Chr3LG5_183228002	2016 Rosthern	2.70E-04	0.47	0.07	
		2017 Rosthern	1.94E-04	0.42	0.08	
		2013 Sutherland	4.47E-05	0.50	0.09	0.08
	Chr5LG3_288274354	2017 Rosthern	1.46E-04	0.43	0.09	
		2017 Sutherland	2.56E-04	0.42	0.08	0.08
	Chr5LG3_436433014	2016 Rosthern	4.70E-04	0.47	0.07	
		2017 Rosthern	5.68E-04	0.41	0.07	0.07
	Chr6LG2_50477045	2016 Sutherland	5.73E-05	0.37	0.11	
		2017 Sutherland	8.79E-05	0.44	0.09	0.10
	Chr6LG2_372463590	2017 Rosthern	1.78E-05	0.45	0.11	
		2013 Sutherland	1.84E-05	0.51	0.10	0.11
	Chr7LG7_7724682	2016 Rosthern	9.12E-04	0.46	0.06	
		2017 Rosthern	8.69E-05	0.43	0.09	
		2016 Sutherland	1.56E-04	0.35	0.10	
		2017 Sutherland	3.18E-04	0.42	0.08	0.08
Starch Conc. (5)	Chr2LG1_457185	2016 Rosthern	2.13E-04	0.45	0.08	
		2017 Rosthern	1.33E-04	0.43	0.09	
		2013 Sutherland	6.75E-05	0.54	0.08	
		2017 Sutherland	5.32E-04	0.43	0.07	0.08
	Chr3LG5_175219020	2016 Rosthern	5.55E-04	0.44	0.07	
		2013 Sutherland	7.48E-04	0.52	0.06	
		2017 Sutherland	7.27E-04	0.43	0.07	0.06
	Chr3LG5_197482300	2016 Rosthern	8.18E-05	0.46	0.09	
		2016 Sutherland	1.11E-03	0.51	0.05	
		2017 Sutherland	2.81E-04	0.44	0.08	0.07
	Chr3LG5_234519042	2016 Rosthern	3.70E-04	0.44	0.07	
		2013 Sutherland	1.47E-04	0.53	0.07	
		2016 Sutherland	1.61E-03	0.51	0.05	
		2017 Sutherland	7.51E-04	0.43	0.07	0.06
	Chr5LG3_436433014	2016 Rosthern	3.81E-04	0.44	0.07	
		2017 Rosthern	5.58E-04	0.42	0.07	
		2017 Sutherland	3.70E-04	0.44	0.07	0.07
	Chr7LG7_223388467	2016 Rosthern	4.37E-04	0.44	0.07	
		2016 Sutherland	6.48E-04	0.52	0.06	
		2017 Sutherland	6.98E-04	0.43	0.07	0.06
	Chr7LG7_8229439	2016 Rosthern	8.92E-04	0.43	0.06	
		2017 Rosthern	7.60E-04	0.41	0.07	
		2016 Sutherland	3.38E-04	0.52	0.06	
		2017 Sutherland	2.06E-04	0.44	0.08	0.07
	Chr7LG7_486526644	2016 Rosthern	1.18E-04	0.45	0.09	
		2017 Rosthern	2.48E-04	0.42	0.08	
		2017 Sutherland	1.54E-04	0.45	0.08	0.08
	Sc03839_38033	2017 Rosthern	5.47E-04	0.42	0.07	
		2016 Sutherland	3.02E-04	0.53	0.06	
		2017 Sutherland	5.70E-04	0.43	0.07	0.07
Protein Conc. (5)	Chr3LG5_138253621	2016 Rosthern	1.95E-03	0.47	0.05	
		2017 Rosthern	1.25E-03	0.59	0.04	
		2013 Sutherland	5.03E-04	0.56	0.05	0.05
	Chr3LG5_194530376	2017 Rosthern	5.10E-05	0.62	0.07	
		2013 Sutherland	5.54E-04	0.56	0.05	
		2016 Sutherland	2.46E-05	0.68	0.06	
		2017 Sutherland	9.22E-04	0.45	0.06	0.06
	Chr5LG3_145264443	2016 Rosthern	5.35E-04	0.49	0.06	
		2017 Rosthern	1.29E-03	0.59	0.04	0.05

The number indicated in parenthesis after the name of each trait represents the number of trials the trait was measured. Only markers which were significant in multiple trials for a given trait are listed in the table. In each SNP ID, Chr and LG refers to chromosome and linkage group followed by the base pair position. For non-chromosomal SNPs, Sc and and SSC refers to scaffold and superscaffold followed by the scaffold number and base pair position. Each locus is represented by one SNP marker of the LD block. ^†^R-square value presented is the difference of R-square explained by the model with and without SNP.

**Figure 5:**
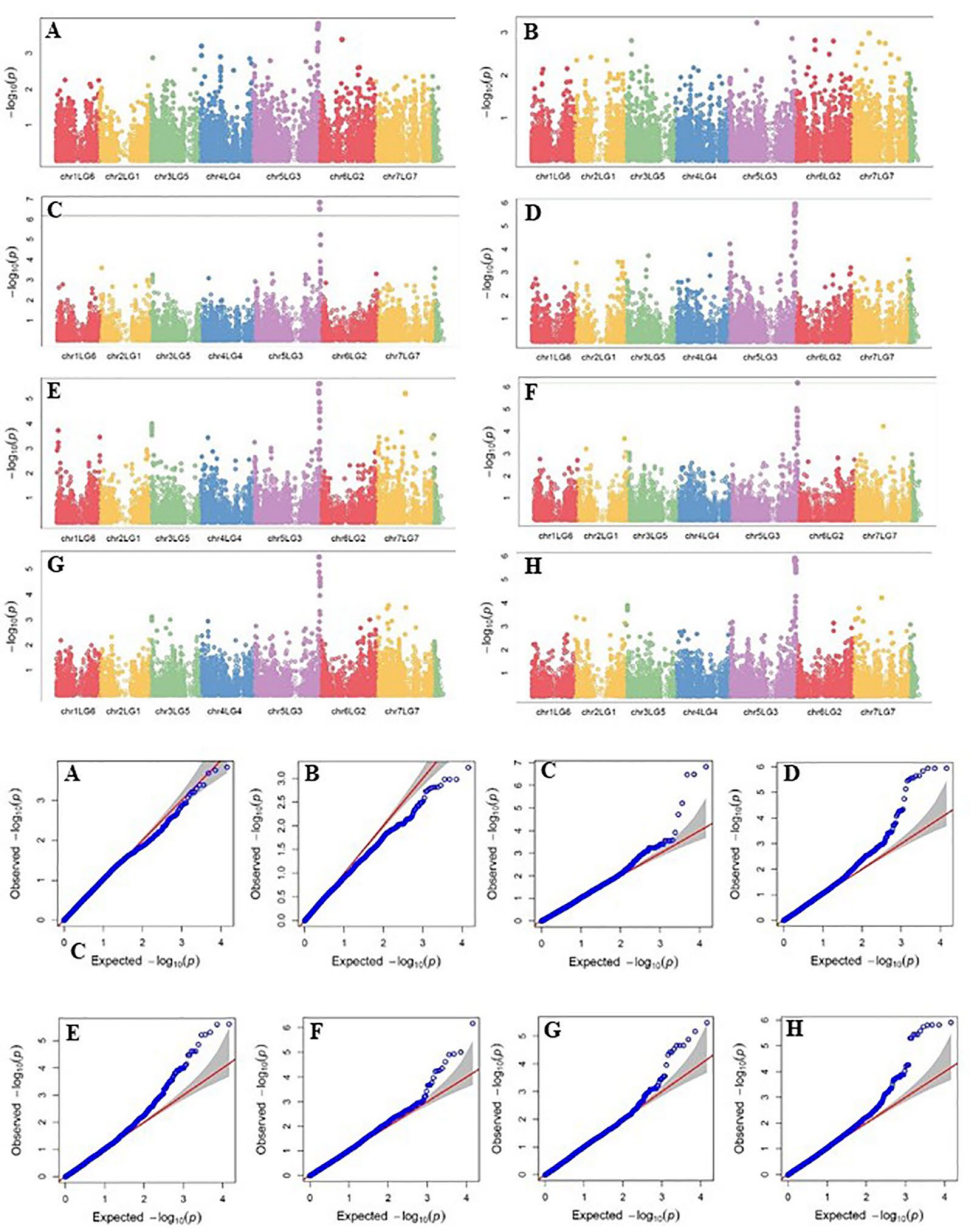
Manhattan plots and the corresponding Q-Q plots representing the identification of SNP markers associated with plant height in multiple trials. **(A)** 2013 Fargo **(B)** 2013 Sutherland, **(C)** 2014 Sutherland, **(D)** 2015 Sutherland, **(E)** 2016 Rosthern, **(F)** 2016 Sutherland, **(G)** 2017 Rosthern, and **(H)** 2017 Sutherland. The Manhattan plots are based on association of 15608 chromosomal and1269 non-chromosomal SNPs with plant height of 135 pea accessions in the multi-year, multi-locational trials.

**Figure 6:**
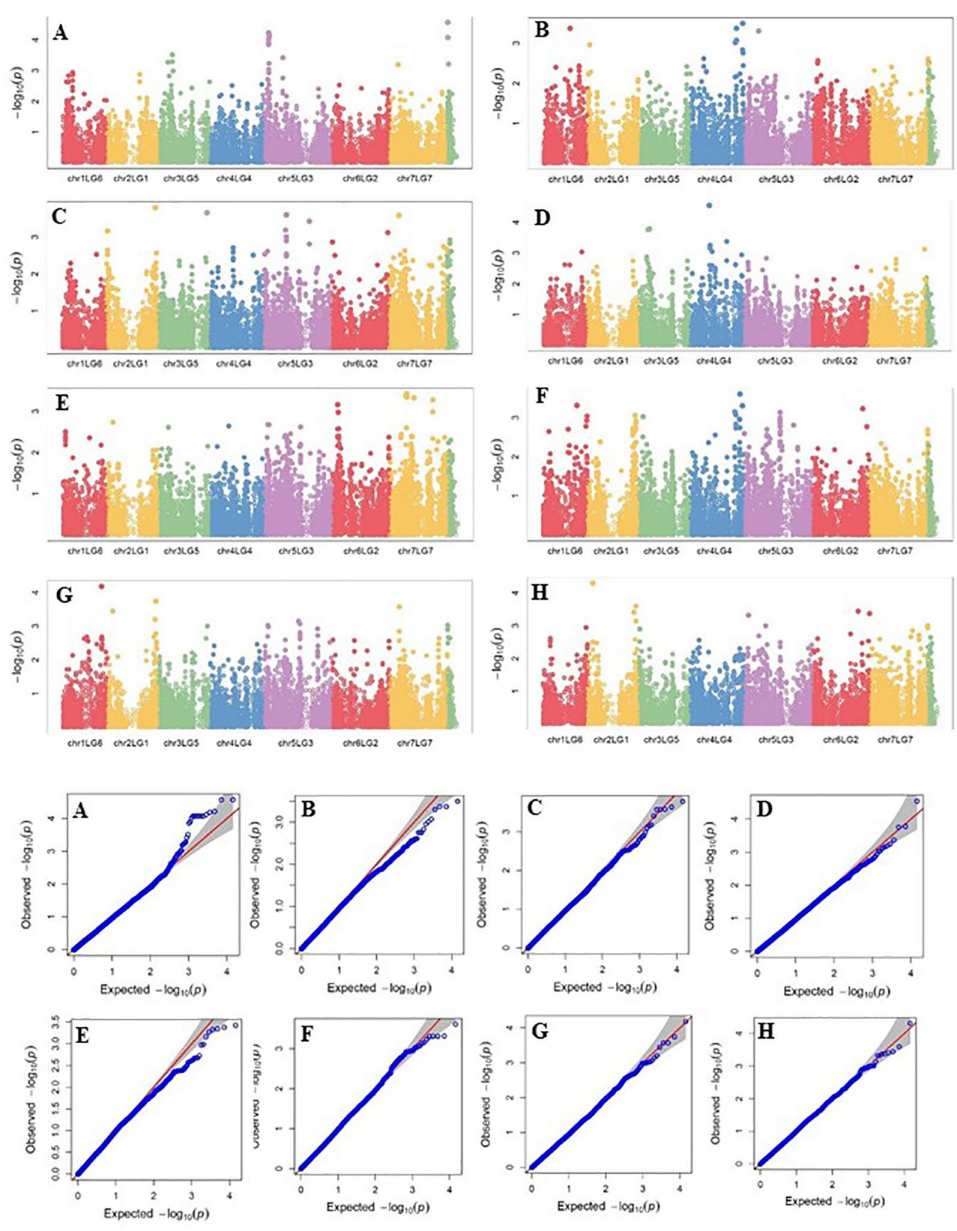
Manhattan plots and the corresponding Q-Q plots representing the identification of SNP markers associated with lodging resistance in multiple trials. **(A)** 2013 Fargo, **(B)** 2014 Fargo, **(C)** 2014 Sutherland, **(D)** 2015 Fargo, **(E)** 2015 Sutherland, **(F)** 2016 Rosthern, **(G)** 2016 Sutherland, and **(H)** 2017 Sutherland. The Manhattan plots are based on association of 15608 chromosomal and 1269 non-chromosomal SNPs with the lodging score measured on a 1-9 rating scale (1 = upright to 9 = completely lodged) in 135 pea accessions in the multi-year, multi-locational trials.

Four SNP markers on chromosome 5 were associated with plant height in four to seven of the nine trials. The R-square value of a model with SNP ranged up to 0.72. Five SNP markers associated with lodging resistance were positioned on chromosomes 1, 2, 3 and 5. SNP marker Chr2LG1_409403647 was identified in four of the 10 trials. Manhattan plots showing the association of SNP markers with plant height and lodging resistance in multiple trials, and the corresponding Q-Q plots are presented as examples from this research in **Figure 5** and **Figure 6**, respectively. The Q-Q plots represent the observed P values of each SNP marker against the expected P values. The Manhattan plots in **Figure 5** show the significant association of SNP markers on chromosome 5 (LG3) with plant height in each of the individual trials presented. The Manhattan plots in **Figure 6** show the significant association of SNP markers on multiple chromosomes with lodging resistance. In all the Q-Q plots of lodging resistance (**Figure 6**), the observed P values are almost the same as expected values.

Two SNP markers on chromosome 1, Chr1LG6_57305683 and ChrLG6_366513463, were associated with yield in three of the 10 trials. Four SNP markers were associated with seed weight, of which SNP marker Chr1LG6_176606388 is located on chromosome 1, and three other SNP markers were positioned on non-chromosomal scaffolds.

Seven SNP markers associated with two seed morphological traits, seed shape and seed dimpling, were identified. Four markers associated with seed shape are distributed on chromosomes 2, 5 and 7, and were associated with seed shape in three to four of the six trials. Two markers on chromosome 1 and one marker on chromosome 3 were associated with seed dimpling. SNP marker chr1LG6_100615820 was associated with seed dimpling in four of the six trials.

Multiple SNP markers were associated with four of the seed quality traits including concentrations of seed acid detergent fibre (ADF), neutral detergent fiber (NDF), starch and protein. Five SNP markers on chromosomes 5, 6 and 7, and eight markers on chromosomes 2, 3, 5, 6 and 7 were identified to be associated with ADF and NDF, respectively. Two markers Chr6LG2_372463590 and Chr7LG7_7724682 were common for ADF and NDF concentrations. Multiple markers positioned on chromosomes 2, 3, 5 and 7 were associated with seed starch concentration, of which three markers Chr2LG1_457185, Chr3LG5_234519042, and Chr7LG7_8229439 were associated with starch concentration in four of the five trials. Two SNP markers on chromosome 3 and one marker on chromosome 5 are associated with seed protein concentration. Chr3LG5_138253621 and Chr3LG5_194530376 are associated with protein concentration in three and four of the five trials, respectively.

Of all the MTAs that were observed in ≥50% of the trials, the following markers explained the highest average phenotypic variance (PV) across the traits: Sc00936_29805 (8% PV) and Sc03817_83023 (8% PV) for DTF, Chr3LG5_112288560 (7% PV) and Chr3LG5_126657675 (6% PV) for DTM, Chr5LG3_566189651 (9% PV), Chr5LG3_572899434 (9% PV) and Chr5LG3_573518168 (8% PV) for plant height, Chr3LG5_197482300 (10% PV) and Chr6LG2_68264764 (10% PV) for seed shape, Chr1LG6_46289124 (6% PV) and Chr1LG6_100615820 (6% PV) for seed dimpling, Chr7LG7_7724682 (8% PV) as a common marker for both ADF and NDF, Chr2LG1_457185 (8% PV) and Chr7LG7_486526644 (8% PV) for seed starch concentration, and Chr3LG5_194530376 (6% PV) for seed protein concentration.

## Discussion

With the availability of cost-effective, high throughput SNP genotyping methods and genomic resources, GWAS has been used as an effective method to identify alleles associated with traits of many crop species including legumes (Desgroux et al., 2016; Sun et al., 2017; Mourad et al., 2018). The current GWAS was undertaken to identify SNP markers associated with several important field pea breeding traits. The natural diversity of pea accessions selected in the 23 pea breeding programs across the world was used to identify trait-linked SNP markers, which could potentially be used for MAS in pea breeding programs. The pea accessions used in this study include accessions from pea breeding programs in Africa, Asia, Australia, Europe and North America, which represent the genetic variations used in these breeding programs as genetic sources for multiple traits. These accessions were expected to possess a diversity of alleles for various agronomic and seed quality traits, and thus were selected for this GWAS study to identify loci controlling multiple traits.

GBS identified 16,877 good quality SNPs, of which 15,609 were distributed across seven chromosomes of pea and 1268 were non-chromosomal SNPs. LD patterns of population structure are important for association mapping (Flint-Garica et al., 2003), thus we analyzed the LD of 135 GWAS accessions by chromosome. The LD decay estimates of the 7 pea chromosomes varied from 0.03 to 0.18 Mb. Siol et al. (2017) reported that LD decays steeply in pea, and the median r^2^ value was less than 0.05 at a genetic distance of ∼3 cM. The clustering of 135 accessions into nine major groups (K = 9) partially independent of their geographical origin reflects the use by pea breeders of genetic variation from diverse sources. Siol et al. (2017) grouped 917 *Pisum* accessions into 16 clusters of which spring and winter accessions represented 10 and 4 clusters, respectively.

The genetic diversity represented by the pea GWAS panel was used for identification of MTAs. In a previous GWAS study of pea, using 175 pea accessions and genotyping based on a 13.2K SNP array, Desgroux et al. (2016) detected 52 loci associated with *Aphanomyces* root rot resistance which included novel loci that validated the reported major and minor QTLs. They also confirmed the linkage between *Aphanomyces* resistance alleles and late flowering alleles, and reported the break of linkage between resistance alleles and colored flowers.

The traits selected for this GWAS study included agronomic traits (DTF, DTM, lodging resistance, seed yield and seed weight), seed characteristics (seed dimpling and seed shape) and seed quality traits (fiber, protein and starch concentrations), all of which are important targets for pea breeding globally. We identified QTLs for all of these traits in our previous study (Gali et al., 2018) using multiple recombinant inbred line (RIL) populations derived from bi-parental crosses. The current research is expected to expand the understanding of genetic loci governing these traits. Genetic relatedness (or kinship) and population structure are known as the major confounding factors that may lead to spurious associations in GWAS (Yu et al., 2006). Thus, upon verification of Q-Q plots, we used MLM method with the combination of Q and K matrices for association analysis, which has been used for association analysis in many plant species (Hao et al., 2012; Huang and Han, 2014).

Using the pea GWAS panel, MTAs were identified for all the traits in repeated tests. Flowering time is one of the key determinants of pea adaptation to different ecological and geographical regions, thus the pea GWAS panel is an ideal population for identification of loci controlling flowering time. Over 20 loci related to flowering time and inflorescence development have been identified in pea and the interactions of these loci determine the flowering time, of which *HIGH RESPONSE* (*HR*), *STERILE NODES* (*SN*), and *LATE FLOWERING* (*LF*) loci are important (reviewed by Weller and Ortega, 2015). In the pea GWAS panel, we identified nine loci for flowering time and five loci for maturity time in repeated tests illustrating the diverse nature of the panel.

Major and minor QTLs were identified for plant height in pea in previous studies. Tar'an et al. (2003) reported three major QTLs and Hamon et al. (2013) identified three minor QTLs. Gali et al. (2018) identified a major QTL for plant height on LG3, in three RIL populations, which explained 33-65% of the phenotypic variance. Ferrari et al. (2016) also reported QTL for plant height on LG3. Similarly, in the pea GWAS panel, we identified four loci on chromosome 5 (LG3) associated with plant height. These four loci together represented a region of ∼7.5 million base pairs on chromosome 5 and previously reported SNP marker Psc7220p181 (Gali et al., 2018) is in proximity of this locus. The pea GWAS panel has greater genetic variation for plant height, compared to the RIL populations, with over 3-fold difference between minimum and maximum plant height. Thus, by capturing the diversity for this trait in the GWAS panel, the major loci for plant height were confirmed to be on chromosome 5 (LG3).

Major QTLs explaining 58% (Tar'an et al., 2003), 50% (Smitchger 2017), and >30% of phenotypic variance for lodging resistance were identified in bi-parental mapping populations (Gali et al., 2018). Ferrari et al. (2016) identified QTLs for lodging resistance on LG3 and LG4. In the current GWAS study, in addition to a locus on chromosome 5 (LG3), additional loci on chromosomes 1, 2, and 3 (LGs 6, 1 and 5) were also identified for association with lodging resistance. Identification of these additional loci could be due to the wide range of diversity for lodging resistance in the GWAS panel as the individual accessions ranged from a lodging score of 1.0 to 9.0 on a 1-9 rating scale. Co-localization of QTLs of plant height and lodging resistance was reported in previous studies (Tar'an et al., 2003; Gali et al., 2018), but in the current study the loci associated with these two traits were not co-localized.

We identified two loci on chromosome 1 (LG6) for association with grain yield in three of the ten trials conducted using the pea GWAS panel. The locus represented by the SNP marker Chr1LG6_366513463 was also associated with DTF. In previous studies based on RILs, multiple QTLs for grain yield were identified on multiple linkage groups (Krajewski et al., 2012; Gali et al., 2018; Tar'an et al., 2004). Since the genetic variation for grain yield is contributed by many loci each contributing a minor portion of the variance for this trait, or largely affected by GxE interactions, it is possible that in the pea GWAS panel we could not identify multiple loci for this trait in repeated tests.

Using the pea GWAS panel, four loci were identified for association with seed weight. One of these loci is on chromosome 1 (LG6) and the other three are located on scaffolds that couldn't be positioned on the assembled chromosomes. In comparison, we earlier reported major QTLs for seed weight on LG3, LG4 and LG6 (Gali et al., 2018). For seed dimpling, two loci on chromosome 1 (LG6) and one locus on chromosome 3 (LG5) were associated with the trait in repeated tests, as compared to the identified key locus on LG5 (Gali et al., 2018). The loci identified for seed shape in repeated trials were positioned on chromosomes 3, 6 and 7 (LGs 5, 2 and 7, respectively), and supports the earlier reported major QTLs on LG2 and LG5 (Gali et al., 2018). In the current study, the four SNP markers identified for association with seed shape were also associated with either seed starch or fiber concentrations.

For all the seed quality traits tested, i.e. seed starch, fiber and protein concentrations, multiple associated markers distributed on different chromosomes were identified. Markers distributed on chromosomes 2, 3, 5 and 7 (LGs 1, 5, 3 and 7) were associated with seed starch concentration. Loci for this trait are known to be positioned on LGs 2, 5 and 7 in PR-07 mapping population (Gali et al., 2018). The markers associated with acid and neutral detergent fiber concentrations were on chromosomes 2, 3, 5, 6 and 7. These traits are known to be controlled by multiple loci (Gali et al., 2018). SNP markers associated with seed protein concentration were on chromosomes 3 (LG5) and 5 (LG3). QTLs for seed protein concentration on LG3 are known in PR-07 mapping population and the loci identified on chromosome 3 (LG5) are additional. Overall, this GWAS study identified new MTAs for seed quality traits.

Overall, detection of multiple MTAs in the GWAS panel compared to RIL populations is as expected because of the ability to detect a range of genes controlling the phenotype in this panel, while QTL detection in RIL populations is limited to the alleles segregating from the two parents. The increased resolution in the GWAS panel is also a result of the historical recombination in this panel, rather than the more limited recombination in the progeny of a bi-parental population. Overall the SNP markers identified in this study often corresponded to the loci reported for the same traits at the linkage group level. However, the current markers differed from the reported markers when compared for base pair position within the same linkage group and did not represent the exact same locus. The identified MTAs are valuable for pea breeders to identify sources of genetic variation for these traits. The average phenotypic variance explained by identified MTAs is ≤10%, and it has to be noted that most agronomic traits are controlled by multiple genes each with minor effect.

Some of the trait-linked markers identified in this study using diverse germplasm are useful to validate the QTL regions identified in earlier studies up to the linkage group level. The sequences of flanking markers of previously reported QTLs (Gali et al., 2018) were used to identify the corresponding regions in the pea genome assembly used in this study. Other than one QTL for plant height, the markers identified in this study were different than the previously reported QTLs in comparison of base pair positions, though they were on the same linkage group. This is possibly because of the greater phenotypic diversity in the GWAS population than in the previous bi-parental populations. We will validate the markers identified in this study with those identified in earlier studies both by genotyping and *in silico* experiments in future studies and explore the candidate genes within the genomic regions of identified loci.

In this study, we performed a GWAS to detect genome regions controlling quantitative traits, using 16,877 SNP markers in a genetically diverse panel of 135 pea germplasm accessions. We identified multiple significant loci associated with agronomic and seed traits of pea. SNP markers identified for association with plant height (Chr5LG3_566189651 and Chr5LG3_572899434), lodging resistance (Chr2LG1_409403647) yield (Chr1LG6_57305683 and Chr1LG6_366513463), seed weight (Chr1LG6_176606388), seed starch concentration (Chr2LG1_457185, Chr3LG5_234519042 and Chr7LG7_8229439), and seed protein concentration (Chr3LG5_194530376) can be of potential use for marker-assisted selection in future pea breeding. The loci identified in this study can be used for further analysis to identify the causal gene(s), to select genetic variation, for marker-assisted trait introgression, as well to pyramid multiple genes in pea through marker-assisted breeding. The genotypic data should be a useful resource for the detection of other agriculturally important loci for many other traits using association analysis.

## Data Availability Statement

The datasets generated for this study can be found in the ENA https://www.ebi.ac.uk/ena/browser/view/PRJEB35147.

## Author Contributions

TW, BT, and KG conceptualized the study. AS, KM, MH, AM, PS, RM, and CD conducted the field trials for phenotyping of GWAS panel. TW and AS co-ordinated the trials at different locations. ET conducted the statistical analysis for phenotypic data. KG genotyped the GWAS panel. KG and VL conducted genotypic and association analysis. KG drafted the manuscript with suggestions from TW. All authors contributed to the manuscript review.

## Funding

Funding for this research from Saskatchewan Ministry of Agriculture and Saskatchewan Pulse Growers is gratefully acknowledged.

## Conflict of Interest

Author VL was employed by company AgriGenome Labs Pvt. Ltd., Hyderabad, India.

The remaining authors declare that the research was conducted in the absence of any commercial or financial relationships that could be construed as a potential conflict of interest.
